# Calprotectin Is a Circulating Biomarker and Potential Therapeutic Target for Sarcopenia in Chronic Obstructive Pulmonary Disease

**DOI:** 10.1002/jcsm.70196

**Published:** 2026-01-25

**Authors:** Liwei Liao, Jiaye Li, Weidong Xu, Yan Yin, Zilin Wang, Chang Li, Yanxia Li, Xiaoming Zhou, Mingming Deng, Gang Hou

**Affiliations:** ^1^ National Center for Respiratory Medicine, State Key Laboratory of Respiratory Health and Multimorbidity, National Clinical Research Center for Respiratory Diseases, Institute of Respiratory Medicine, Chinese Academy of Medical Sciences, Department of Pulmonary and Critical Care Medicine, Center of Respiratory Medicine China‐Japan Friendship Hospital Beijing China; ^2^ China‐Japan Friendship Hospital (Institute of Clinical Medical Sciences) Chinese Academy of Medical Sciences & Peking Union Medical College Beijing China; ^3^ Department of Pulmonary and Critical Care Medicine First Hospital of China Medical University Shenyang China; ^4^ Respiratory Department The First Affiliated Hospital of Dalian Medical University Dalian China; ^5^ Respiratory Department, Center for Pulmonary Vascular Diseases, Fuwai Hospital, National Center for Cardiovascular Diseases, Chinese Academy of Medical Sciences Peking Union Medical College Beijing China

**Keywords:** chronic obstructive pulmonary disease (COPD), paquinimodcalprotectin, sarcopenia

## Abstract

**Background:**

Sarcopenia, an important complication of chronic obstructive pulmonary disease (COPD), is significantly associated with increased mortality. Systemic inflammation is an important trigger of COPD‐related skeletal muscle dysfunction. Calprotectin is a damage‐associated molecular pattern involved in the inflammatory response, but its exact role and mode of action in COPD‐related skeletal muscle dysfunction remain unclear. This study aimed to determine whether calprotectin is involved in COPD‐related sarcopenia.

**Methods:**

In this study, 235 patients with stable COPD were divided into the development (*n* = 117) and validation (*n* = 118) groups, and serum calprotectin concentrations were measured by enzyme‐linked immunosorbent assays (ELISAs). Paquinimod, an oral calprotectin‐specific inhibitor, was used to investigate the involvement of calprotectin in cigarette smoke (CS)‐induced skeletal muscle dysfunction in vivo.

**Results:**

Handgrip strength and quadriceps muscle strength, essential indicators of muscle strength, were negatively correlated with serum calprotectin levels (*r* = −0.367, *p* < 0.001; *r* = −0.409, *p* < 0.001). The 5‐time sit‐to‐stand test results, which reflect endurance and physical strength, were positively correlated with serum calprotectin levels (*r* = 0.290, *p* = 0.006). Ultrasound measurement of the rectus femoris muscle revealed negative correlations of serum calprotectin levels with both muscle thickness (*r* = −0.448, *p* < 0.001) and cross‐sectional area (*r* = −0.495, *p* < 0.001). Furthermore, serum calprotectin levels were significantly greater in patients with sarcopenia than in those without sarcopenia (90.09 ± 25.72 ng/mL vs. 59.56 ± 23.22 ng/mL, *p* < 0.001). Importantly, serum calprotectin levels could effectively predict sarcopenia in COPD patients in the development set (AUC = 0.811) and validation set (AUC = 0.805). In C57BL/6 mice with CS‐induced muscle dysfunction, paquinimod (10 mg/kg/day) reduced CS‐induced muscle mass loss (skeletal muscle weight 1.15% ± 0.09% vs. 1.33% ± 0.09%; *p* = 0.005) and increased the muscle cross‐sectional area (1375 ± 536.9 μm^2^ vs. 2094 ± 470.2 μm^2^; *p* < 0.001). Paquinimod also reduced CS‐induced muscle weakness, as indicated by increased grip strength (214.9 ± 31.38 g vs. 333.1 ± 34.93 g; *p* < 0.01). Paquinimod inhibited ubiquitin–proteasome system activity, reduced protein degradation marker levels, attenuated oxidative stress and increased antioxidant enzyme levels in CS‐exposed mice.

**Conclusions:**

Serum calprotectin levels can be used to accurately predict sarcopenia in patients with COPD, and the calprotectin inhibitor paquinimod is a potential treatment for CS‐induced skeletal muscle dysfunction.

## 1 Introduction

Chronic obstructive pulmonary disease (COPD) is a common chronic respiratory disease with limitations in airflow and is preventable and treatable [1]. COPD is often accompanied by systemic diseases, such as cardiovascular disease, osteoporosis, depression and anxiety [2]. Sarcopenia is characterized by decreased muscle strength and mass [3], with a prevalence rate of 15%–55% [4] in patients with COPD. Patients with stable COPD and sarcopenia have elevated dyspnoea index scores and decreased exercise endurance [5]. Moreover, reduced exercise performance and low activity levels contribute to muscle depletion and atrophy, exacerbating the vicious cycle of disease development.

Systemic inflammation is a significant risk factor for common comorbidities in patients with COPD [6]. Systemic inflammation refers to a condition in which irritants, such as cigarette smoke (CS), induce inflammation and oxidative stress components in patients with COPD, which ‘spill over’ into the circulatory system via the bloodstream, manifesting as systemic inflammation. Previous studies [7] have shown that systemic inflammation is closely associated with skeletal muscle function in patients with COPD [8]. Moreover, studies have indicated that the levels of the inflammatory mediators tumour necrosis factor (TNF)‐α, interleukin (IL)‐6, IL‐8 and C‐reactive protein are elevated in patients with stable COPD and are related to decreased skeletal muscle function [9]. Further research suggests that sarcopenia in patients with COPD is strongly correlated with systemic inflammation; among inflammatory mediators, serum TNF‐α levels are a decisive factor in patients with COPD and sarcopenia [10]. During acute exacerbations of COPD, systemic inflammation peaks and accelerates acute muscle loss [11]. These findings suggest that systemic inflammation could play a crucial role in the development of both COPD and sarcopenia. Thus, therapies aimed at reducing the inflammatory response may be beneficial for ameliorating muscle pathology in patients with COPD.

Calprotectin, a 24‐kDa heterodimeric protein, is a damage‐associated molecular pattern composed of S100A8 and S100A9 subunits belonging to the S100 calcium‐binding protein family [12]. Calprotectin is abundantly expressed in neutrophils, macrophages, and other immune cells and plays a crucial role in innate immunity. Calprotectin participates in a signalling cascade mediated by toll‐like receptor 4 (TLR4) and/or the receptor for advanced glycation end‐products (RAGE) [13]. The pathophysiological significance of calprotectin in respiratory diseases, including cystic fibrosis, asthma and pulmonary infectious diseases, has been demonstrated in preclinical animal models and clinical studies [14]. S100A9 (a subunit of calprotectin) signalling plays a key role in the progression of COPD caused by smoking and aging. Blocking S100A9 signalling could protect against lung damage and inflammation in patients with COPD and age‐related lung decline [15]. Nevertheless, the exact role and mode of action of calprotectin in COPD‐related skeletal muscle dysfunction remain unclear.

This study investigated the role of calprotectin in COPD‐related sarcopenia. The clinical value of serum calprotectin level as a screening tool for the detection of sarcopenia among patients with COPD was evaluated in two independent cohorts. In addition, we explored the potential therapeutic value of paquinimod (a specific inhibitor of calprotectin) in skeletal muscle dysfunction induced by cigarette smoking.

## 2 Methods

### 2.1 Human Participants

Between August 2018 and December 2019, patients who were diagnosed with stable COPD were recruited from two Chinese hospitals: the First Affiliated Hospital of China Medical University and the First Affiliated Hospital of Dalian Medical University. The inclusion criteria were a diagnosis of stable COPD on the basis of the criteria of the Global Initiative for Chronic Obstructive Lung Disease (GOLD) guidelines and an age in the range of 40–80 years. The exclusion criteria were recent COPD exacerbation within the past month, COPD complicated with coronary heart disease, active lung disease (asthma, lung cancer, active tuberculosis, bronchiectasis or diffuse lung disease), current oral steroid or antibiotic treatment or inability to read or understand the informed consent form. A total of 117 enrolled individuals from the First Hospital of China Medical University formed the development set for assessing the clinical significance of calprotectin and determining cutoff values. An additional 118 patients from the First Hospital of Dalian Medical University constituted the validation set. Ethical approval was obtained from the research ethics committees of the First Hospital of China Medical University (No. 2018‐144‐2) and the First Hospital of Dalian Medical University (PJ‐KS‐KY‐2022‐348(X)).

### 2.2 Clinical Data Collection

Measurements were collected at a single time point, during which we recorded the participants' general characteristics, including age, sex and smoking status. Spirometry was performed in compliance with the standards set forth by the American Thoracic Society and the European Respiratory Society, utilizing the Jaeger MasterScreen system (Jaeger, Viasys Healthcare GmbH, Hoechberg, Germany). The modified British Medical Research Council dyspnoea score [16] and the COPD Assessment Test [17] were utilized to evaluate dyspnoea symptoms and health status.

Exercise tolerance was evaluated using the 6‐min walking distance (6MWD) implemented in compliance with protocols established by the American Thoracic Society in 2002. For this purpose, participants were directed to walk as extensively as possible along a straight, enclosed corridor measuring 30 m for 6 min.

### 2.3 Measurement of Serum Calprotectin Levels

Blood samples were obtained, and serum was isolated by centrifugation at 1000 × g for 15 min. Then, the serum was stored at −80°C until further analysis. Serum calprotectin was detected using enzyme‐linked immunosorbent assays (ELISAs) following the manufacturer's instructions (Bühlmann Laboratories AG, Schőnenbuch, Switzerland).

### 2.4 Measurement Thickness and Cross‐Sectional Area of the Musculus Rectus Femoris

In accordance with previous studies, ultrasound was performed to measure skeletal muscle mass (SMM) [18]. SMM was assessed using grayscale ultrasound scanning conducted with an Aixplorer ultrasound scanning system equipped with a 4‐ to 15‐MHz linear array transducer. Participants were instructed to rest quietly for 15 min prior to examination and then assume a supine position on the examination table with complete muscular relaxation throughout the body. All the sonographers involved had received standardized training and had more than 10 years of clinical experience in musculoskeletal imaging. To minimize external force–induced muscle deformation, a customized stabilizing bracket was employed to maintain perpendicular alignment of the ultrasound probe relative to the participant's dominant leg. The transducer was precisely positioned at 3/5 of the distance between the anterior superior iliac spine and the superior patellar border (the thickest portion of the thigh). This standardized positioning ensured consistent visualization of the rectus femoris cross‐section within a single imaging plane across all participants while excluding other quadriceps muscles from the field of view. The scanning depth was optimized to visualize the femoral orientation. Prior to image acquisition, gentle contraction–relaxation manoeuvres were performed to delineate the intermuscular septa. Following image freezing, the medial echogenic border of the rectus femoris was manually traced using a movable cursor to calculate both the muscle thickness (RF_thick_) and cross‐sectional area (RF_csa_). Three consecutive measurements with ≤ 10% intermeasurement variability were obtained, with their mean values adopted as final quantitative outcomes for subsequent analysis.

### 2.5 Assessment of Sarcopenia

The definition of sarcopenia in this study aligned with the guidelines established by the Asian Working Group for Sarcopenia [19]. Sarcopenia is characterized by three key components. The first is low muscle mass, indicated by the skeletal muscle mass index (SMMI, < 7.0 kg/m^2^ for males and < 5.7 kg/m^2^ for females), which is the SMM divided by height squared. The second is low muscle strength as assessed by handgrip strength (HGS, < 7.0 kg/m^2^ for men and < 5.7 kg/m^2^ for women), and the third is poor physical performance defined as a 5‐time chair stand test duration of ≥ 12 s. Muscle mass was measured using bioelectrical impedance analysis with the InBody770 device (InBody, Seoul, Korea) following the training manual. Skeletal muscle mass was estimated by a commercial BIA system that uses the multifrequency‐based proprietary algorithms of the InBody770 device, the validity of which has been supported by previous studies [20, 21]. Participants were asked to remove as much of their clothing as possible, stand barefoot on the device, align their heels/feet with the electrodes and wait for their weight to be measured. Next, they were required to grip hand electrodes with arms slightly extended, not touching the armpits, for results. HGS was evaluated in terms of adherence to a standardized technique by using a JAMAR Plus+ hand dynamometer (Sammons Preston, Bolingbrook, IL, USA). During the HGS assessment, participants were seated with their elbows flexed at a 90° angle while maintaining a neutral wrist position. They were instructed to squeeze with maximum force in three consecutive attempts, with a 30‐s rest between measurements. Additionally, physical performance was assessed using the five‐time chair stand (5STS) test. In the 5STS test, the time taken for an individual to stand up and sit down from a chair five times as quickly as possible was recorded. Patients were initially screened for possible sarcopenia using either low handgrip strength or a prolonged 5STS time. Those who met either of these criteria subsequently underwent bioelectrical impedance analysis to assess muscle mass. A confirmed diagnosis of sarcopenia was made only when low muscle strength (or poor physical performance) was accompanied by a low skeletal muscle mass index (SMMI).

### 2.6 Mouse Model of CS Exposure

All animal experiments conducted in this study were approved by the Animal Ethics Committee of China‐Japan Friendship Hospital. In terms of CS‐induced muscle dysfunction, the proportion of male patients is much greater than that of female patients [22]. In previous studies, male mice were most commonly used [23, 24]. Thus, male C57BL/6J mice aged 8–10 weeks were procured from Huafukang Biotechnology Company. To expose the mice to CS, a dedicated barrier facility was utilized with a systemic exposure system (8050II; Hepu, Tianjin, China). Following a previously established method [23], the mice were exposed to 20 Marlboro cigarettes (Philip Morris, Stamford, CT, USA) per session twice daily, 6 days a week, for 3 months. Paquinimod (ABR‐25757, T7310, Targetmol, Shanghai, China) was administered orally at 10 mg/kg/day for 6 weeks. This paquinimod dosage was selected on the basis of the relevant literature [25]. For paquinimod treatment, six mice were allocated to each of the 4 final experimental groups (*n* = 6 per group; total *n* = 24). C57BL/6 mice were randomly divided into two main exposure groups: one exposed to CS and the other to filtered air (air) for a duration of 12 weeks. At the 6‐week time point, mice in each exposure group were further randomly subdivided to receive either paquinimod (10 mg/kg/day) or an equivalent volume of placebo via oral gavage for the remaining 6 weeks.

Mouse grip strength was assessed using a grip strength meter (YLS‐13A, Yanyi, China) at the end of the CS exposure period. Each mouse gripped the instrument grids and was gently pulled back by its tail until the mouse released its grip. The force meter recorded the maximum exerted force, and grip strength was calculated as the average of three measurements.

Fresh muscles were immediately fixed in 4% paraformaldehyde. Paraffin embedding, dehydration and haematoxylin and eosin (H&E) staining were performed on muscle sections. Randomly selected images of H&E‐stained muscle were used to measure the cross‐sectional area (CSA) of the muscle fibres. ImageJ software (US NIH, Bethesda, MD, USA) was used to analyse the CSA, and a minimum of 50 myofibres were evaluated in each mouse. The muscle CSA in each group is given as a percentage relative to that of the controls.

### 2.7 Western Blotting

Western blotting analysis was conducted as previously described [26]. The primary antibodies used were a rabbit polyclonal antibody against GAPDH (1:1000; Cell Signalling Technology, 5174), a mouse monoclonal antibody against atrogin‐1 (1:1000; Santa Cruz Biotechnology, sc‐166 806), a mouse monoclonal antibody against MuRF1 (1:1000; Santa Cruz Biotechnology, sc‐398 608), a rabbit monoclonal antibody against Nrf2 (1:1000; Cell Signalling Technology, 12721), and a rabbit monoclonal antibody against HO‐1 (1:1000; Cell Signalling Technology, 26416).

### 2.8 Statistical Analysis

All data are presented as the mean ± standard deviation and were analysed by SPSS 13.0 (SPSS Corp., Chicago, IL, USA). The normal distribution of the data was evaluated using histograms, quantile–quantile plots and the Shapiro–Wilk test. For normally distributed data, differences between two groups were evaluated using *t* tests, and nonnormally distributed data were analysed using Mann–Whitney tests. Multiple group comparisons were conducted using analysis of variance, with subsequent application of the Tukey–Kramer correction for handling multiple comparisons when the data exhibited a normal distribution. Conversely, the Kruskal–Wallis test was employed for data that did not follow a normal distribution, accompanied by Dunn's multiple comparison test. Multiple linear regression was conducted to confirm the associations between calprotectin levels and the identified clinical features. The Pearson correlation coefficient was used to evaluate correlations. Partial correlation analyses were performed as a method for correction, with BMI, age and sex serving as covariates and serum calprotectin level as the independent variable. Statistical significance was set at *p* < 0.05.

## 3 Results

### 3.1 Association of Circulating Calprotectin Levels With Skeletal Muscle Dysfunction in Participants With COPD

To evaluate the clinical relevance of calprotectin, circulating serum calprotectin levels were quantified by ELISAs in participants diagnosed with COPD. The characteristics of our participants are shown in Table 1. Multiple linear regression revealed a significant effect of calprotectin on multiple clinical variables (Table S1), including the 6MWD, mMRC, CAT, BFR, FFMI, SMMI, RFthick, RFcsa, QMS, 5STS and HGS (Table S1). Specifically, the levels of calprotectin were still significantly correlated with muscle mass and mass strength indicators, such as SMMI, RFthick, RFcsa, QMS, 5STS and HGS. However, calprotectin levels were not significantly correlated with the BFR or FFMI after the inclusion of FEV_1_%. These results suggest that although COPD is associated with a general state of malnutrition or even cachexia, calprotectin might be a more specific indicator of skeletal muscle dysfunction than of fat loss.

**TABLE 1:** Baseline characteristics of patients in the development and validation groups.

Variable\group	Training group (*n* = 117)	Validation group (*n* = 118)	*p*
Age (years)	64.9 ± 12.0	63.9 ± 9.2	0.110
Sarcopenia	43	40	0.668
Smoking status	—	—	0.848
Current	32	29	—
Former	73	74	—
Never	12	15	—
Sex (M/F)	83/34	77/41	0.349
Pulmonary function	—	—	—
FEV_1_ (L)	1.6 ± 0.6	1.5 ± 0.5	0.370
FEV_1_% pred (%)	59.8 ± 21.0	56.2 ± 20.7	0.368
FVC (L)	2.8 ± 0.9	2.7 ± 0.8	0.452
FVC% pred (%)	82.5 ± 23.7	84.5 ± 20.0	0.700
FEV_1_/FVC (%)	55.2 ± 9.9	53.1 ± 11.1	0.232
GOLD stage	—	—	—
Stage I	19	21	0.986
Stage II	59	57	—
Stage III	30	31	—
Stage IV	9	9	—
6MWD (m)	369.0 ± 74.2	359.1 ± 78.1	0.370
5STS (s)	7.8 ± 3.3	7.4 ± 2.3	0.624
BMI (kg/m^2^)	23.9 ± 3.9	23.6 ± 3.8	0.532
Body fat (%)	28.9 ± 6.3	29.0 ± 6.4	0.907
FFM (kg)	47.0 ± 9.3	46.3 ± 9.6	0.735
FFMI (kg/m^2^)	16.8 ± 4.2	16.7 ± 2.3	0.173
SMM (kg)	17.7 ± 4.4	17.1 ± 4.2	0.737
SMMI (kg/m^2^)	6.4 ± 1.1	5.9 ± 1.4	0.143
Grip strength (kg)	26.6 ± 8.1	27.0 ± 1.2	0.787

Abbreviations: 5STS, 5‐repetition sit‐to‐stand; 6MWD, 6‐min walk distance; FEV_1_, forced expiratory volume in 1 s; FEV_1_% pred, FEV_1_ percent predicted; FFM, fat‐free mass; FFMI, fat‐free mass index; FVC, forced vital capacity; FVC% pred, FVC percent predicted; SMM, skeletal muscle mass; SMMI, skeletal muscle mass index.

**FIGURE 1:**
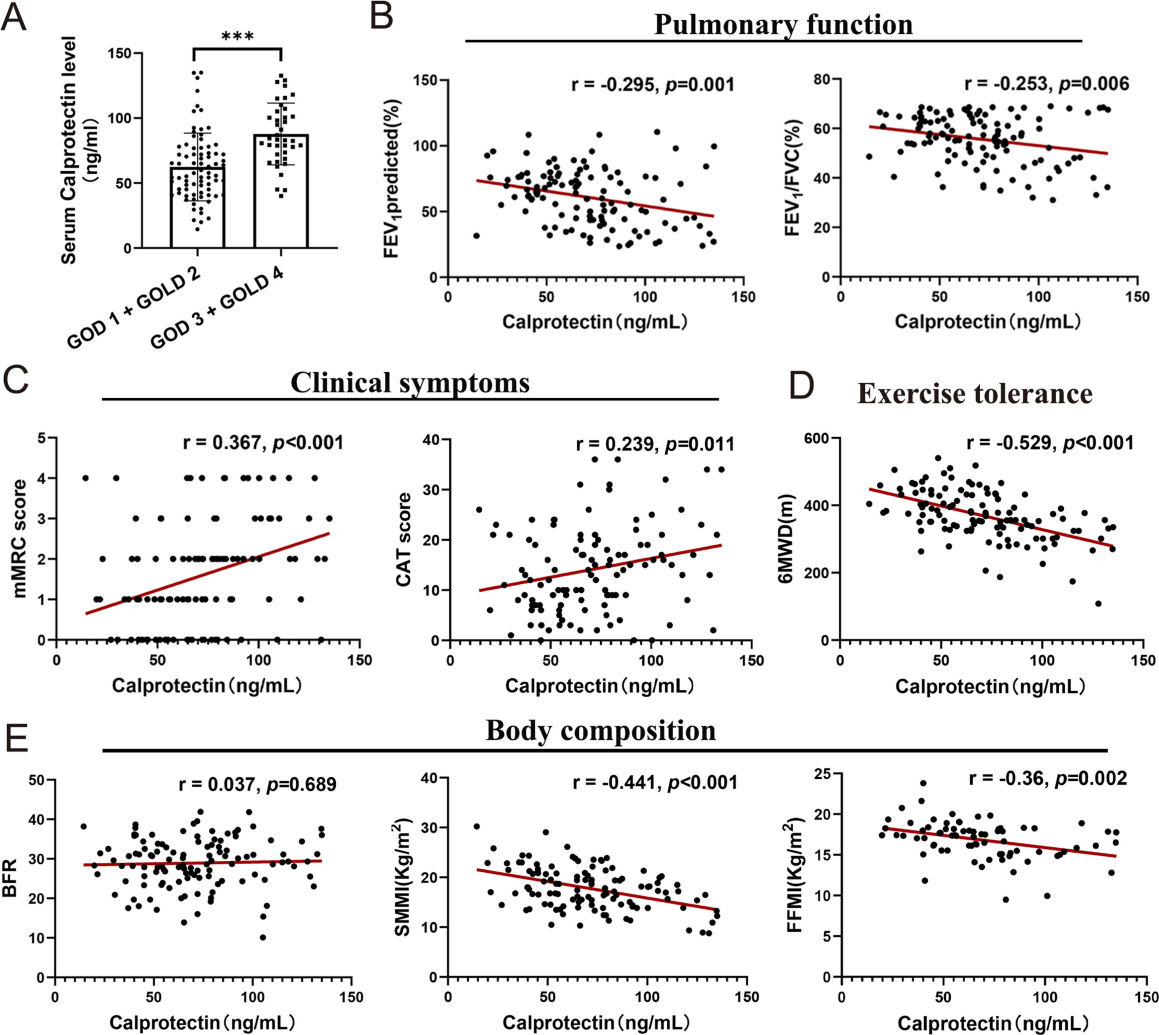
Relationships between serum calprotectin levels and clinical features of patients with COPD. (A) Differences in serum calprotectin levels between patients with GOLD stage 1 or GOLD stage 2 and patients with GOLD stage 3 or GOLD stage 4 disease. (B) Relationships between serum calprotectin levels and pulmonary function, including FEV_1_% predicted and FEV_1_/FVC. (C) Relationships between serum calprotectin levels and clinical symptoms, including the mMRC and CAT scores. (D) Relationship between serum calprotectin levels and 6MWD. (E) Relationships between serum calprotectin levels and BMI, SMMI, and FFMI. FEV_1_% predicted, forced expiratory volume in the first second percentage predicted. BMI, body mass index; CAT, COPD assessment test; FFMI, fat‐free mass index; FVC% predicted, forced vital capacity percentage predicted; GOLD, global initiative for chronic obstructive lung disease; mMRC, modified British Medical Research Council; SMMI, skeletal muscle mass index; 6MWD, 6‐min walking distance.

Initially, we observed increased calprotectin expression in participants classified as having GOLD stage 3 or 4 disease compared with those categorized as having GOLD stage 1 or 2 disease (Figure 1A). Furthermore, correlations were identified between calprotectin levels and pulmonary function parameters. Specifically, serum calprotectin levels were negatively correlated with the predicted forced expiratory volume in 1 s (FEV_1_) (*R* = −0.295, *p* = 0.001) and the FEV_1_/forced vital capacity (FVC) ratio (*R* = −0.253, *p* = 0.006) (Figure 1B). Additionally, calprotectin levels were related to worse clinical symptoms and health conditions. Notably, serum calprotectin levels were significantly negatively correlated with the modified Medical Research Council dyspnoea score (*R* = 0.367, *p* < 0.001) and COPD assessment test score (*R* = 0.239, *p* < 0.001) (Figure 1C). With respect to exercise tolerance represented by the 6MWD, higher serum calprotectin levels were linked to a lower 6MWD (*R* = −0.529, *p* < 0.001) (Figure 1D). Moreover, we explored the relationship between circulating serum calprotectin levels and body composition. Our findings revealed a negative correlation between serum calprotectin levels and the fat‐free mass index (*R* = −0.36, *p* = 0.002), whereas no significant relationship was observed between serum calprotectin levels and the body fat ratio (*R* = 0.037, *p* = 0.689). Finally, concerning skeletal muscle function, serum calprotectin levels were negatively correlated with SMM (*R* = −0.441, *p* < 0.001) (Figure 1E). Overall, these results emphasize the potential of calprotectin as a significant biomarker in patients with COPD.

### 3.2 Clinical Value of Circulating Calprotectin Levels for Differentiating Sarcopenia in Patients With COPD

**FIGURE 2:**
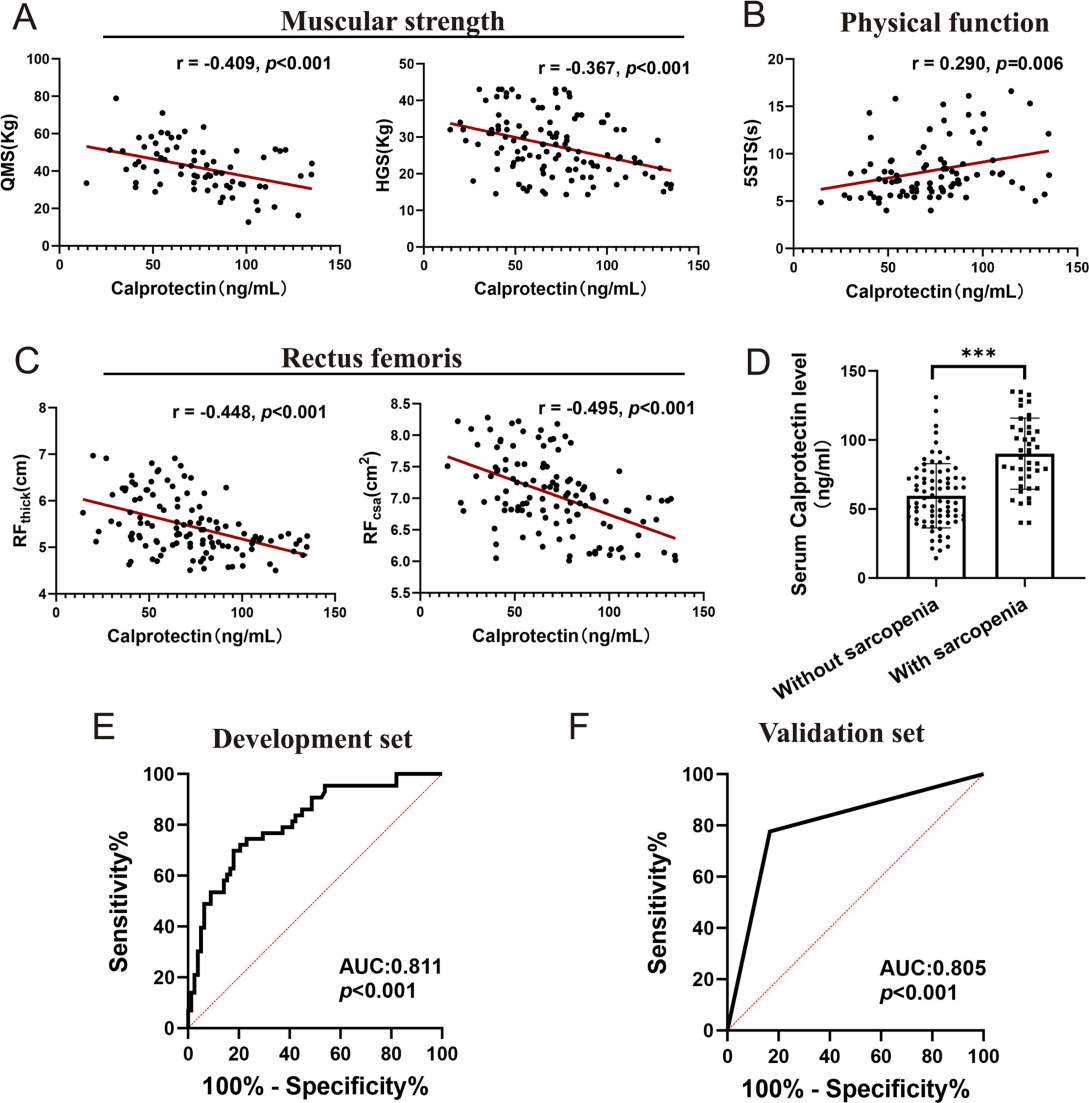
Associations of serum calprotectin levels with skeletal muscle function and the predictive value of serum calprotectin levels for sarcopenia in patients with COPD. (A) Relationships between serum calprotectin levels and muscular strength (QMS, HGS). (B) Relationship between serum calprotectin levels and physical function (5STS). (C) Relationships between serum calprotectin levels and RF_thick_ and RF_csa_. (D) Difference in serum calprotectin levels between patients with and without sarcopenia. (E) Receiver operating characteristic curve analysis of serum calprotectin levels for the prediction of sarcopenia in the development set. (F) Receiver operating characteristic curve analysis of serum calprotectin levels for the prediction of sarcopenia in the validation set. HGS, handgrip strength; QMS, quadriceps muscle strength; RF_thick_, thickness of the rectus femoris; RF_csa_, cross‐sectional area of the rectus femoris; 5STS, five‐time chair sit‐to‐stand.

The association between circulating serum calprotectin levels and skeletal muscle function was investigated in patients with COPD. Two essential indicators of muscle strength, HGS and quadriceps muscle strength (QMS), were negatively correlated with serum calprotectin levels (HGS: *R* = −0.367, *p* < 0.001; QMS: *R* = −0.409, *p* < 0.001) (Figure 2A). The 5STS time, a measure of lower‐limb functional performance, was positively associated with serum calprotectin levels (*R* = 0.290, *p* = 0.006) (Figure 2B), suggesting that a higher calprotectin concentration was associated with poorer exercise performance. Ultrasound measurements of the musculus rectus femoris revealed negative correlations between serum calprotectin levels and both muscle thickness (*R* = −0.448, *p* < 0.001) and CSA (*R* = −0.495, *p* < 0.001) (Figure 2C). These findings show that the serum calprotectin level can serve as a biomarker for assessing skeletal muscle function in patients with COPD.

Additionally, the diagnostic significance of the serum calprotectin level in differentiating sarcopenia in patients with COPD, following the guidelines from the Asian Working Group for Sarcopenia, was assessed. The presence of sarcopenia in patients with COPD was associated with significantly higher calprotectin levels than those in patients without sarcopenia (90.09 ± 25.72 ng/mL vs. 59.56 ± 23.22 ng/mL, *p* < 0.001) (Figure 2D). Receiver operating characteristic curves generated from the development set demonstrated the ability of serum calprotectin to predict sarcopenia. Using a cutoff value of 78.45 ng/mL, serum calprotectin had a sensitivity of 69.77% and a specificity of 82.05% in differentiating sarcopenia. The area under the curve (AUC) was calculated as 0.811 (Figure 2E). These findings were validated in an independent validation set, with an AUC of 0.805 (*p* < 0.001) for differentiating sarcopenia using calprotectin (Figure 2F). Overall, these results strongly suggest that serum calprotectin expression can act as a valuable biomarker for predicting sarcopenia in patients with COPD.

### 3.3 Elevation of Circulating Calprotectin Levels in a Preclinical Mouse Model of COPD

**FIGURE 3:**
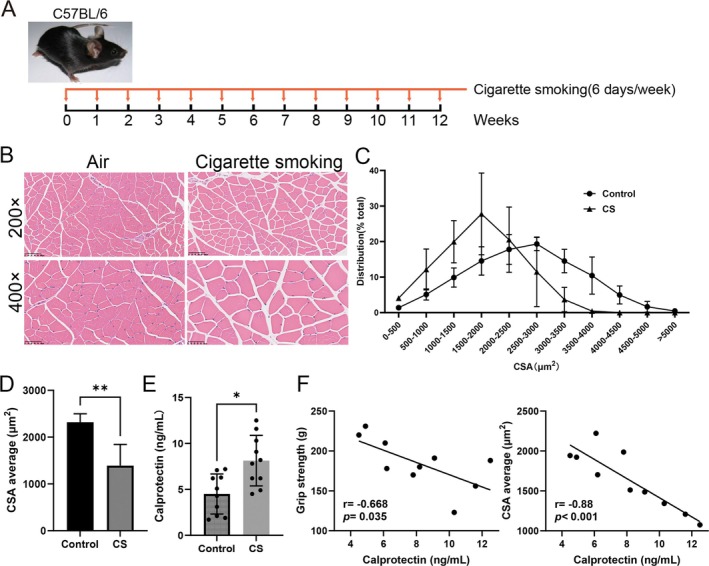
Elevation of circulating calprotectin levels in cigarette smoke‐exposed mice. (A) Process of modelling CS‐exposed mice. (B) Representative H&E staining of myofiber cross‐sections of the Gast and comparison of healthy mice and CS‐exposed mice in terms of the cross‐sectional area of Gast muscle fibres. (C and D) Distribution and quantification of myofiber diameter in the Gast from the different groups. (E) Elevated circulating calprotectin levels in CS‐exposed mice. (F) Correlations of calprotectin levels with grip strength (left) and muscle cross‐sectional area (right) in CS‐exposed mice. *p < 0.05; **p < 0.01; ***p < 0.001.

To validate the clinical findings, we established a mouse model of COPD by exposing the mice to CS (Figure 3A). After 12 weeks of CS exposure, histological examination using H&E staining revealed that CS exposure significantly reduced the CSA of muscle fibres (Figure 3B–D). The circulating levels of calprotectin substantially increased after 12 weeks of CS exposure in mice (Figure 3E). Correlative analyses further revealed strong negative associations between circulating calprotectin levels and grip strength, similar to the CSA of muscle fibres, in CS‐exposed mice (Figure 3F). These collective findings indicate that elevated levels of calprotectin are correlated with impaired skeletal muscle function in the context of CS exposure associated with COPD.

### 3.4 Calprotectin Blockade With Paquinimod Protects Mice Against Skeletal Muscle Dysfunction Induced by Cigarette Smoking

**FIGURE 4:**
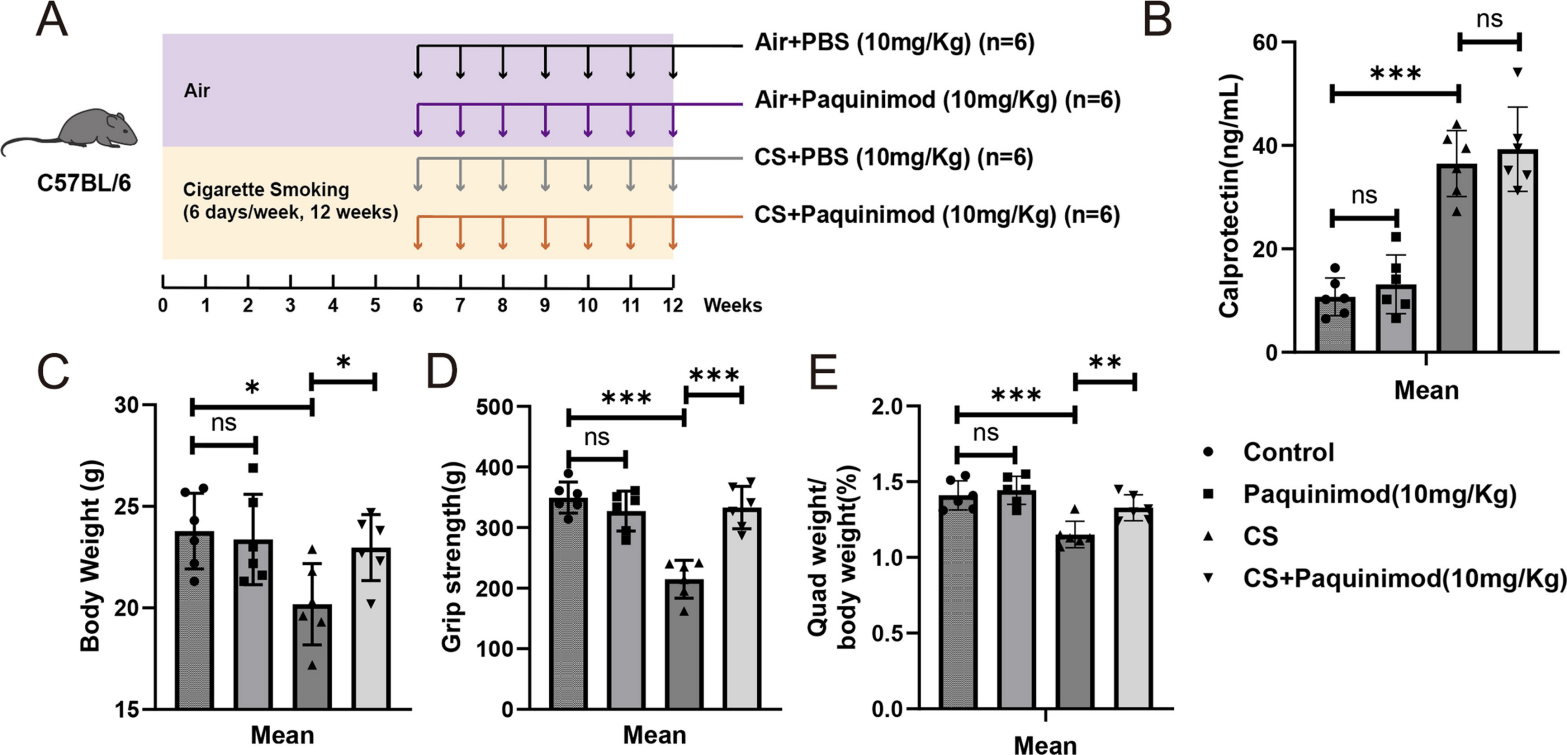
Paquinimod inhibited calprotectin activity and protected mice against cigarette smoke‐induced skeletal muscle dysfunction. (A) Schematic diagram of the intraperitoneal administration of paquinimod (paquinimod; 10 mg/kg/day; *n* = 10) or vehicle (PBS every week; *n* = 10) in the CS‐exposed mouse model. (B) Serum calprotectin levels in the different groups. (C–E) Comparisons of body weight, grip strength and relative quadriceps (Quad) weight among control mice, control mice treated with paquinimod, CS‐exposed mice, and CS‐exposed mice treated with paquinimod. *p < 0.05; **p < 0.01; ***p < 0.001.

To validate the therapeutic potential of calprotectin in preventing CS‐induced skeletal muscle dysfunction, we established a CS‐exposed mouse model and utilized paquinimod, a specific calprotectin inhibitor (Figure 4A). First, the effect of paquinimod treatment on calprotectin levels was analysed, and the results revealed no significant effect on serum calprotectin levels (Figure 4B). The therapeutic potential of paquinimod was subsequently evaluated in the CS‐exposed mouse model. After 3 months of CS exposure, the weight loss of the mice was effectively reversed by paquinimod treatment (Figure 4C). The impairment of grip strength caused by CS exposure was mitigated by paquinimod (Figure 4D). Furthermore, paquinimod significantly alleviated the loss of muscle mass caused by CS exposure (Figure 4E). These findings indicate that paquinimod provides protective effects against skeletal muscle dysfunction induced by cigarette smoking in vivo without altering serum calprotectin concentrations.

### 3.5 Paquinimod Ameliorates CS‐Induced Muscle Wasting and Protein Degradation in Mice

**FIGURE 5:**
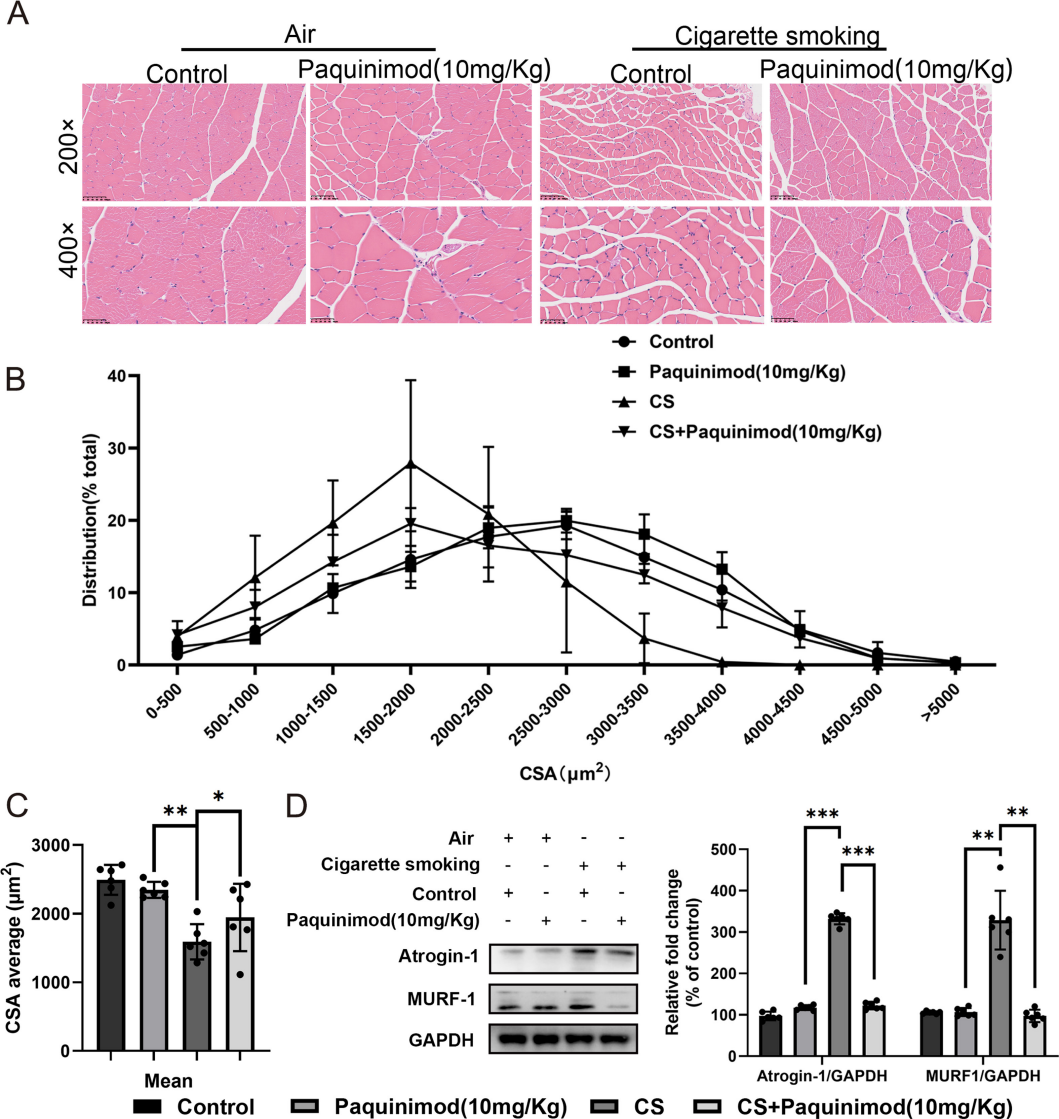
Paquinimod ameliorated muscle wasting and protein degradation in a cigarette smoke‐exposed mouse model. (A) Representative H&E staining of myofiber cross‐sections of the Gast. (B and C) Distribution and quantification of the Gast myofiber diameter in the different groups. (D) Western blot analysis of the levels of muscle‐specific ubiquitin E3 ligases (atrogin‐1 and MuRF1) in each group of mice (left); quantification of atrogin‐1 and MuRF1 expression by Western blot analysis (right). *p < 0.05; **p < 0.01; ***p < 0.001.

The impact of paquinimod on muscle atrophy induced by CS was examined. Histological analysis revealed that the CSA of gastrocnemius (Gast) muscle fibres was notably lower in CS‐exposed mice than in control mice (Figure 5A). However, paquinimod treatment effectively prevented the loss of muscle CSA induced by CS exposure (Figure 5B,C). Western blot analysis confirmed the upregulation of the ubiquitin–proteasome system, including that of muscle‐specific ubiquitin E3 ligases (atrogin‐1 and MuRF1), in CS‐exposed muscles, which was effectively reversed by paquinimod treatment (Figure 5D). These findings highlight the protective role of paquinimod against CS‐induced muscle wasting and protein degradation.

### 3.6 Paquinimod Mitigates the Inflammatory Response and Oxidative Stress in a CS‐Exposed Mouse Model

**FIGURE 6:**
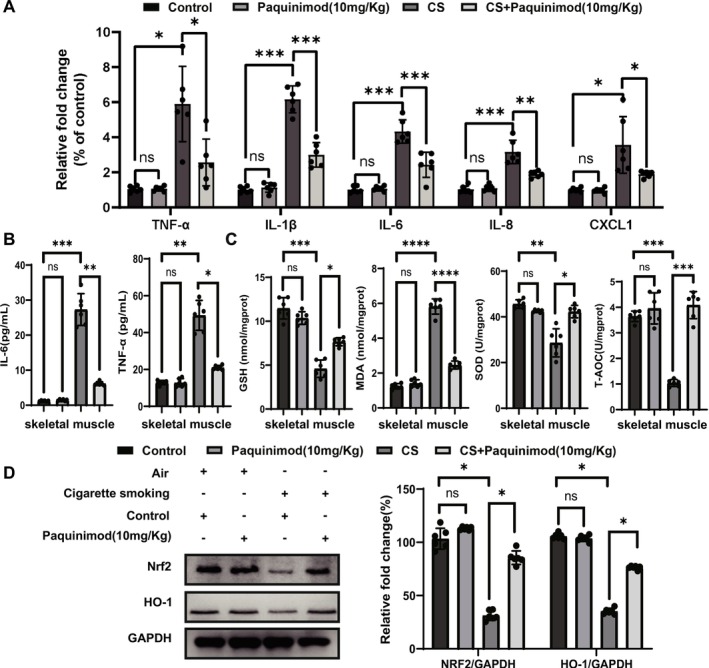
Paquinimod attenuates the inflammatory response and oxidative stress in the cigarette smoke‐exposed mouse model. (A) The mRNA levels of inflammatory factors (TNF‐α, IL‐6, IL‐8, CXCL1, and IL1β) in skeletal muscle measured by qPCR. (B) TNF‐α and IL‐6 levels in different groups measured by ELISAs. (C) Levels of GSH, MDA, SOD2, and T‐AOC in the mouse Gast muscle. (D) Western blot analysis of the levels of antioxidant markers (Nrf2 and HO‐1) in each group of mice. *p < 0.05; **p < 0.01; ***p < 0.001.

Inflammation and oxidative stress play crucial roles in the pathogenesis of skeletal muscle dysfunction. The impact of paquinimod on inflammatory cytokine production was investigated in the CS‐exposed mouse model. CS exposure elevated the mRNA expression levels of proinflammatory factors, such as TNF‐α, IL‐1β, IL‐6, IL‐8 and CXCL1, in skeletal muscle. However, treatment with paquinimod effectively reversed the CS‐induced upregulation of these inflammatory factors (Figure 6A, Table S2). ELISAs confirmed the attenuating effect of paquinimod on the increased secretion of TNF‐α and IL‐6 in CS‐exposed skeletal muscle (Figure 6B). These findings demonstrate that paquinimod exerts a protective effect against skeletal muscle dysfunction by modulating the inflammatory response. Moreover, the activities of antioxidant enzymes, including GSH, SOD2 and T‐AOC, were reduced in muscles exposed to CS but partially recovered following paquinimod administration. Similarly, the increase in the level of MDA (a marker of oxidative stress) in CS‐exposed muscles tended to decrease with paquinimod treatment (Figure 6C). Furthermore, paquinimod reversed the downregulation of key antioxidant stress markers, including Nrf2 and HO‐1, induced by CS (Figure 6D). These results highlight the efficacy of paquinimod in alleviating oxidative stress levels in mice exposed to CS.

## 4 Discussion

Our study identified elevated serum calprotectin levels as a discriminative biomarker and a promising therapeutic target for sarcopenia in patients with COPD. In the present study, ELISA revealed that serum calprotectin levels were notably elevated in patients with COPD and sarcopenia compared with those without COPD and could predict sarcopenia in patients with COPD in independent cohorts. Additionally, the targeted inhibition of calprotectin by paquinimod ameliorated skeletal muscle dysfunction in a preclinical mouse model by modulating oxidative stress and inflammatory pathways. As cigarette smoking is the most common risk factor for the development of COPD, CS‐exposed mice were used in this study to simulate COPD‐related sarcopenia, as described in previous studies [27]. Collectively, these findings suggest that calprotectin represents a viable therapeutic target for COPD‐related sarcopenia.

The predictive performance of serum calprotectin for sarcopenia is comparable to that of other proposed biomarkers in COPD, with the added advantage of being pharmacologically targetable. Inflammatory mediators and myokines are potential biomarkers of COPD‐related sarcopenia [28]. For example, plasma levels of the myokines irisin and brain‐derived neurotrophic factor (BDNF) and soluble tumour necrosis factor II (TNF‐II) receptor are reduced in patients with COPD. Plasma levels of Dickkopf‐3 (Dkk‐3) and c‐terminal agrin fragment‐22 (CAF22) can suggest a sarcopenia phenotype in elderly individuals with respiratory diseases [29]. In previous studies, we and others have identified biomarkers such as growth differentiation factor 15 (GDF15) [30] and lipoprotein‐associated phospholipase A2 (Lp‐PLA2) [31] as predictors of sarcopenia in patients with COPD, with reported AUCs of approximately 0.827 and 0.744, respectively. In comparison, serum calprotectin levels demonstrated comparable predictive performance, with AUCs of 0.811 (development set) and 0.805 (validation set). However, among these biomarkers, few are directly targetable by drugs. Therefore, more research is needed to identify predictive and therapeutically targetable biomarkers for sarcopenia in patients with COPD.

The biological rationale for targeting calprotectin in COPD is supported by its established role in driving inflammation and its clinical correlation with disease severity. Calprotectin is an inflammatory protein that is mainly secreted by immune cells, especially neutrophils and macrophages. In COPD, calprotectin signalling affects the infiltration of neutrophils into the BALF [32]. In addition, calprotectin affects MMP‐9 production by macrophages [33]. In inflammatory bowel disease (IBD), calprotectin levels are typically elevated. Measurements of faecal or blood calprotectin levels are widely used in the diagnosis of IBD [34]. Prior investigations have consistently reported increased levels of circulating calprotectin in stable patients with COPD than in healthy individuals. Moreover, calprotectin has been recognized as an independent prognostic indicator for elevated all‐cause mortality in this population [35]. Elevated calprotectin levels are negatively correlated with lung function in patients experiencing acute exacerbations of COPD (AE‐COPD), suggesting a potential link between serum calprotectin levels and progression of AE‐COPD; thus, calprotectin is proposed to be useful as a biomarker for the diagnosis of AE‐COPD [36]. Our study revealed that serum calprotectin levels increased with increasing COPD GOLD score, which is consistent with the findings of previous reports [37]. Importantly, our data indicated that serum calprotectin levels could be used to effectively identify sarcopenia in patients with COPD, establishing a potential diagnostic cutoff value.

Paquinimod (ABR215757), a specific calprotectin inhibitor, has emerged as a clinically translatable candidate for addressing COPD‐related sarcopenia. The management of skeletal muscle dysfunction in patients diagnosed with COPD relies primarily on pulmonary rehabilitation training. However, not all individuals experience improvements in muscle function solely through rehabilitation. Paquinimod is an orally administered drug classified as quinoline‐3‐carboxamide and exhibits immunomodulatory properties [38]. In previous studies on septic acute kidney injury [38] and neuroinflammation [39], paquinimod effectively reduced oxidative stress, which is also a significant factor contributing to skeletal muscle dysfunction in patients with COPD [40]. Paquinimod has obtained the orphan drug designation from the US Food and Drug Administration and has acknowledged safety profiles determined in phase 1 and 2 clinical trials for conditions such as systemic lupus erythematosus [41] and systemic sclerosis [42]. Recent data demonstrate the tolerability of paquinimod in humans. To date, there have been no COPD‐specific trials of paquinimod, although paquinimod decreased lung inflammation, alleviated alveolar destruction and improved lung function in a murine model of COPD [15]. Therefore, paquinimod has therapeutic potential for COPD and holds promise for future human applications.

The therapeutic effects of paquinimod are likely mediated through its broad antagonism of the proinflammatory signalling pathways activated by calprotectin. Although the precise mechanism of action of paquinimod remains incompletely elucidated, it is known to bind S100A9 (a component of the calprotectin heterodimer). This binding prevents calprotectin from engaging its receptors, toll‐like receptor 4 (TLR4) and the receptor for advanced glycation end products (RAGE), thereby modulating downstream signalling without directly affecting calprotectin expression. Our results support this mechanism, demonstrating that paquinimod effectively attenuates CS‐induced skeletal muscle dysfunction in mice without altering serum calprotectin levels. In a prior study modelling COPD, paquinimod was evaluated in both preventive and therapeutic regimens. This intervention was shown to reduce airway inflammation, suppress the release of MCP‐1, IL‐6, KC, MMP‐3 and MMP‐9 and inhibit phosphorylation of ERK and c‐RAF in CS‐induced and age‐related models. Additionally, paquinimod reduced the activation of pathogenic transgenic natural killer T cells, CD115^+^Ly6C^hi^ monocytes and CD11b^+^F4/80^+^CD206^+^ macrophages [15]. In a Parkinson's disease mouse model, paquinimod treatment downregulated the expression of inflammatory cytokines (IL‐6, IL‐1β and TNF‐α) and suppressed the TLR4/NF‐κB signalling pathway [43]. Similarly, in a model of cardiac dysfunction, paquinimod was shown to inhibit the S100A8/A9–TLR4–NLRP3–IL‐1β signalling axis, thereby counteracting its detrimental effects [44]. Collectively, these findings indicate that paquinimod may exert broad anti‐inflammatory effects by antagonizing the calprotectin‐mediated activation of TLR4 and RAGE signalling, thereby disrupting proinflammatory cascades and showing therapeutic potential across a spectrum of inflammatory and immune‐mediated diseases.

Our study has several limitations. First, its cross‐sectional design necessitates additional prospective studies to validate calprotectin levels as predictive of sarcopenia risk. Second, our study did not involve healthy controls. The association of calprotectin with systemic symptoms in patients with COPD needs to be clarified. Moreover, while our study revealed an association between calprotectin and skeletal muscle dysfunction, the specific mechanisms underlying this relationship remain to be fully elucidated and warrant further investigation.

## 5 Conclusion

**FIGURE 7:**
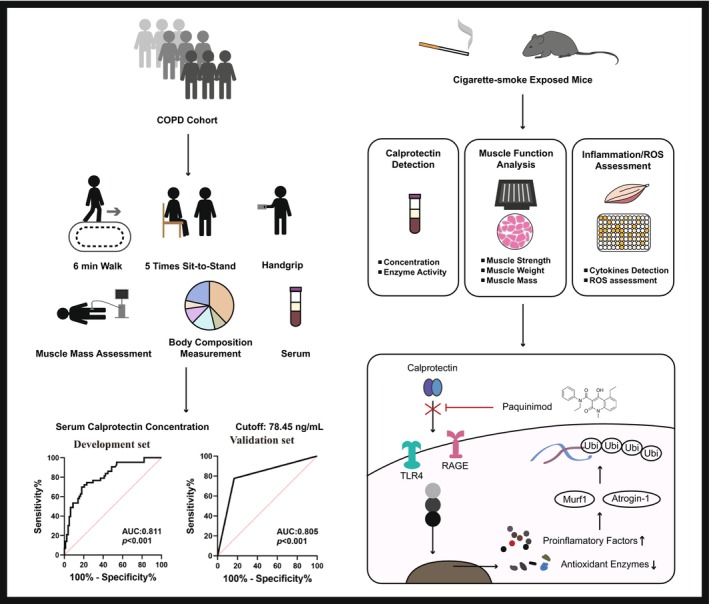
Graphical displays of the study design and main results. Serum calprotectin levels can be used to accurately predict sarcopenia in a COPD cohort, and the calprotectin inhibitor paquinimod could be a potential treatment for CS‐induced skeletal muscle dysfunction.

Our results indicate that serum calprotectin levels can be used to accurately predict sarcopenia in patients with COPD and that the calprotectin inhibitor paquinimod has potential as a treatment for CS‐induced skeletal muscle dysfunction (Figure 7).

## Funding

This research was supported by the National Natural Science Foundation of China (Nos. 82300053, 82570063, and 82400041), the Key Research and Development Program of Heilongjiang (No. 2024ZX12C20), State Key Laboratory of Respiratory Health and Multimorbidity, state Key Laboratory Special Fund 2060204(2024‐QZZZ‐01), and the Fundamental Research Funds for the Central Universities (3332024101).

## Ethics Statement

The inclusion of human participants in our study was approved by the Research Ethics Committee of China Medical University and the Research Ethics Committee of Dalian Medical University. Written informed consent was obtained before the participants participated in our study. Animal experiments were performed according to the National Institutes of Health Animal Use guidelines and experimental protocols were approved by the Animal Ethics Committee of China‐Japan Friendship Hospital.

## Conflicts of Interest

The authors declare no conflicts of interest.

## Supporting information


**Table S1:** Multiple linear regression on calprotectin and clinical variables.
**Table S2:** Mean Ct value of murine skeletal samples detected by qPCR.

**Figure S1:** Successful establishment of a cigarette smoke‐exposed mouse model. (A) H&E staining of the lungs. CS‐exposed mice presented enlargement of the alveolar lumen, parenchymal destruction and alveolar wall rupture. (B) Comparison of control mice and CS‐exposed mice in terms of the mean linear intercept (MLI). (C) Comparison of the mean alveolar area (MAA) between control mice and CS‐exposed mice.

## Data Availability

Data will be made available on request.
