# Supplementary material for: Calprotectin Is a Circulating Biomarker and Potential Therapeutic Target for Sarcopenia in Chronic Obstructive Pulmonary Disease
Source: J Cachexia Sarcopenia Muscle. 2026 Jan 25;17(1):e70196. doi: 10.1002/jcsm.70196 (PMC12833497; doi:10.1002/jcsm.70196)
Supplement: Supplementary file 1 — Table S1: Multiple linear regression on calprotectin and clinical variables. Table S2: Mean Ct value of murine skeletal samples detected by qPCR. [file JCSM-17-e70196-s001.docx]

**STable 1. Multiple linear regression on calprotectin and clinical variables.**

Multiple linear regression models on FEV1% with calprotectin, BMI, gender and age.

| Parameter estimates | Variable | Estimate | Standard  error | 95% CI | \|t\| | P Value | P Value Summary |
| --- | --- | --- | --- | --- | --- | --- | --- |
| β0 | intercept | 68.12 | 17.37 | 33.70 to 102.5 | 3.922 | 0.0002 | *** |
| β1 | Calprotectin | -0.2224 | 0.07098 | -0.3630 to -0.08173 | 3.133 | 0.0022 | ** |
| β2 | BMI | 0.05358 | 0.5106 | -0.9582 to 1.065 | 0.1049 | 0.9166 | ns |
| β3 | gender | -1.879 | 4.339 | -10.48 to 6.719 | 0.4329 | 0.6659 | ns |
| β4 | age | 0.09105 | 0.1643 | -0.2345 to 0.4166 | 0.5541 | 0.5806 | ns |

Multiple linear regression models on FEV1/FVC with calprotectin, BMI, gender and age.

| Parameter estimates | Variable | Estimate | Standard  error | 95% CI | \|t\| | P Value | P Value Summary |
| --- | --- | --- | --- | --- | --- | --- | --- |
| β0 | intercept | 52.52 | 8.009 | 36.65 to 68.38 | 6.557 | <0.0001 | **** |
| β1 | Calprotectin | -0.08944 | 0.03273 | -0.1543 to -0.02459 | 2.733 | 0.0073 | ** |
| β2 | BMI | 0.1590 | 0.2355 | -0.3076 to 0.6255 | 0.6752 | 0.5010 | ns |
| β3 | gender | 3.549 | 2.001 | -0.4155 to 7.514 | 1.774 | 0.0788 | ns |
| β4 | age | 0.1260 | 0.07577 | -0.02415 to 0.2761 | 1.663 | 0.0992 | ns |

Multiple linear regression models on 6MWD with calprotectin, BMI, gender, age and FEV1%.

| Parameter estimates | Variable | Estimate | Standard  error | 95% CI | \|t\| | P Value | P Value Summary |
| --- | --- | --- | --- | --- | --- | --- | --- |
| β0 | intercept | 542.8 | 52.49 | 438.8 to 646.8 | 10.34 | <0.0001 | **** |
| β1 | Calprotectin | -1.261 | 0.2098 | -1.677 to -0.8453 | 6.012 | <0.0001 | **** |
| β2 | BMI | -1.570 | 1.447 | -4.437 to 1.298 | 1.085 | 0.2804 | ns |
| β3 | gender | 24.42 | 12.31 | 0.03273 to 48.80 | 1.984 | 0.0497 | * |
| β4 | age | -1.664 | 0.4663 | -2.588 to -0.7398 | 3.568 | 0.0005 | *** |
| β5 | FEV1% | 0.7198 | 0.2677 | 0.1892 to 1.250 | 2.688 | 0.0083 | ** |

Multiple linear regression models on mMRC with calprotectin, BMI, gender, age and FEV1%.

| Parameter estimates | Variable | Estimate | Standard  error | 95% CI | \|t\| | P Value | P Value Summary |
| --- | --- | --- | --- | --- | --- | --- | --- |
| β0 | intercept | -0.9971 | 1.068 | -3.115 to 1.121 | 0.9334 | 0.3528 | ns |
| β1 | Calprotectin | 0.01509 | 0.004353 | 0.006462 to 0.02372 | 3.467 | 0.0008 | *** |
| β2 | BMI | 0.07797 | 0.02937 | 0.01973 to 0.1362 | 2.654 | 0.0092 | ** |
| β3 | gender | -0.1892 | 0.2505 | -0.6858 to 0.3075 | 0.7552 | 0.4518 | ns |
| β4 | age | 0.01216 | 0.01080 | -0.009252 to 0.03358 | 1.126 | 0.2627 | ns |
| β5 | FEV1% | -0.01685 | 0.005356 | -0.02747 to -0.006230 | 3.146 | 0.0022 | ** |

Multiple linear regression models on CAT with calprotectin, BMI, gender, age and FEV1%.

| Parameter estimates | Variable | Estimate | Standard  error | 95% CI | \|t\| | P Value | P Value Summary |
| --- | --- | --- | --- | --- | --- | --- | --- |
| β0 | intercept | 10.01 | 7.591 | -5.044 to 25.05 | 1.318 | 0.1903 | ns |
| β1 | Calprotectin | 0.07094 | 0.03093 | 0.009623 to 0.1323 | 2.294 | 0.0238 | * |
| β2 | BMI | 0.3277 | 0.2087 | -0.08605 to 0.7415 | 1.570 | 0.1193 | ns |
| β3 | gender | -2.380 | 1.780 | -5.909 to 1.148 | 1.337 | 0.1840 | ns |
| β4 | age | -0.03502 | 0.07674 | -0.1872 to 0.1171 | 0.4563 | 0.6491 | ns |
| β5 | FEV1% | -0.07765 | 0.03805 | -0.1531 to -0.002199 | 2.040 | 0.0438 | * |

Multiple linear regression models on BFR with calprotectin, BMI, gender, age and FEV1%.

| Parameter estimates | Variable | Estimate | Standard  error | 95% CI | \|t\| | P Value | P Value Summary |
| --- | --- | --- | --- | --- | --- | --- | --- |
| β0 | intercept | 4.562 | 3.689 | -2.748 to 11.87 | 1.237 | 0.2188 | ns |
| β1 | Calprotectin | 0.02249 | 0.01474 | -0.006723 to 0.05170 | 1.526 | 0.1300 | ns |
| β2 | BMI | 0.9477 | 0.1017 | 0.7461 to 1.149 | 9.318 | <0.0001 | **** |
| β3 | gender | -7.354 | 0.8649 | -9.068 to -5.640 | 8.503 | <0.0001 | **** |
| β4 | age | 0.1133 | 0.03277 | 0.04834 to 0.1782 | 3.457 | 0.0008 | *** |
| β5 | FEV1% | -0.03346 | 0.01882 | -0.07075 to 0.003830 | 1.778 | 0.0781 | ns |

Multiple linear regression models on FFMI with calprotectin, BMI, gender, age and FEV1%.

| Parameter estimates | Variable | Estimate | Standard  error | 95% CI | \|t\| | P Value | P Value Summary |
| --- | --- | --- | --- | --- | --- | --- | --- |
| β0 | intercept | 4.770 | 0.8713 | 3.032 to 6.508 | 5.474 | <0.0001 | **** |
| β1 | Calprotectin | -0.006113 | 0.003557 | -0.01321 to 0.0009825 | 1.719 | 0.0901 | ns |
| β2 | BMI | 0.5066 | 0.02614 | 0.4544 to 0.5587 | 19.38 | <0.0001 | **** |
| β3 | gender | 1.702 | 0.2382 | 1.227 to 2.177 | 7.147 | <0.0001 | **** |
| β4 | age | -0.01970 | 0.007557 | -0.03478 to -0.004628 | 2.607 | 0.0112 | * |
| β5 | FEV1% | 0.007916 | 0.004614 | -0.001288 to 0.01712 | 1.716 | 0.0907 | ns |

Multiple linear regression models on SMMI with calprotectin, BMI, gender, age and FEV1%.

| Parameter estimates | Variable | Estimate | Standard  error | 95% CI | \|t\| | P Value | P Value Summary |
| --- | --- | --- | --- | --- | --- | --- | --- |
| β0 | intercept | 20.44 | 3.433 | 13.64 to 27.24 | 5.954 | <0.0001 | **** |
| β1 | Calprotectin | -0.06088 | 0.01372 | -0.08807 to -0.03370 | 4.438 | <0.0001 | **** |
| β2 | BMI | 0.05499 | 0.09465 | -0.1326 to 0.2425 | 0.5811 | 0.5624 | ns |
| β3 | gender | -0.4197 | 0.8049 | -2.015 to 1.175 | 0.5215 | 0.6031 | ns |
| β4 | age | -0.009026 | 0.03050 | -0.06946 to 0.05141 | 0.2960 | 0.7678 | ns |
| β5 | FEV1% | 0.02011 | 0.01751 | -0.01459 to 0.05482 | 1.148 | 0.2532 | ns |

Multiple linear regression models on RF_thick_ with calprotectin, BMI, gender, age and FEV1%.

| Parameter estimates | Variable | Estimate | Standard  error | 95% CI | \|t\| | P Value | P Value Summary |
| --- | --- | --- | --- | --- | --- | --- | --- |
| β0 | intercept | 6.563 | 0.4729 | 5.626 to 7.500 | 13.88 | <0.0001 | **** |
| β1 | Calprotectin | -0.008660 | 0.001890 | -0.01241 to -0.004915 | 4.582 | <0.0001 | **** |
| β2 | BMI | -0.01362 | 0.01304 | -0.03946 to 0.01221 | 1.045 | 0.2984 | ns |
| β3 | gender | 0.2121 | 0.1109 | -0.007602 to 0.4318 | 1.913 | 0.0583 | ns |
| β4 | age | -0.01077 | 0.004201 | -0.01910 to -0.002449 | 2.564 | 0.0117 | * |
| β5 | FEV1% | 0.006463 | 0.002413 | 0.001683 to 0.01124 | 2.679 | 0.0085 | ** |

Multiple linear regression models on RF_csa_ with calprotectin, BMI, gender, age and FEV1%.

| Parameter estimates | Variable | Estimate | Standard  error | 95% CI | \|t\| | P Value | P Value Summary |
| --- | --- | --- | --- | --- | --- | --- | --- |
| β0 | intercept | 8.323 | 0.4556 | 7.421 to 9.226 | 18.27 | <0.0001 | **** |
| β1 | Calprotectin | -0.009875 | 0.001821 | -0.01348 to -0.006267 | 5.424 | <0.0001 | **** |
| β2 | BMI | -0.01872 | 0.01256 | -0.04361 to 0.006168 | 1.490 | 0.1389 | ns |
| β3 | gender | 0.1022 | 0.1068 | -0.1095 to 0.3139 | 0.9569 | 0.3407 | ns |
| β4 | age | -0.008589 | 0.004047 | -0.01661 to -0.0005686 | 2.122 | 0.0361 | * |
| β5 | FEV1% | 0.005944 | 0.002324 | 0.001338 to 0.01055 | 2.557 | 0.0119 | * |

Multiple linear regression models on QMS with calprotectin, BMI, gender, age and FEV1%.

| Parameter estimates | Variable | Estimate | Standard  error | 95% CI | \|t\| | P Value | P Value Summary |
| --- | --- | --- | --- | --- | --- | --- | --- |
| β0 | intercept | 49.51 | 11.70 | 26.17 to 72.85 | 4.233 | <0.0001 | **** |
| β1 | Calprotectin | -0.1586 | 0.04739 | -0.2532 to -0.06405 | 3.347 | 0.0013 | ** |
| β2 | BMI | 0.4623 | 0.3363 | -0.2089 to 1.133 | 1.375 | 0.1737 | ns |
| β3 | gender | 9.473 | 2.829 | 3.826 to 15.12 | 3.348 | 0.0013 | ** |
| β4 | age | -0.2732 | 0.09508 | -0.4630 to -0.08342 | 2.873 | 0.0054 | ** |
| β5 | FEV1% | 0.06627 | 0.05743 | -0.04835 to 0.1809 | 1.154 | 0.2526 | ns |

Multiple linear regression models on 5STS with calprotectin, BMI, gender, age and FEV1%.

| Parameter estimates | Variable | Estimate | Standard  error | 95% CI | \|t\| | P Value | P Value Summary |
| --- | --- | --- | --- | --- | --- | --- | --- |
| β0 | intercept | 2.018 | 3.049 | -4.049 to 8.084 | 0.6616 | 0.5101 | ns |
| β1 | Calprotectin | 0.03482 | 0.01257 | 0.009823 to 0.05982 | 2.771 | 0.0069 | ** |
| β2 | BMI | 0.09737 | 0.07976 | -0.06129 to 0.2560 | 1.221 | 0.2256 | ns |
| β3 | gender | -1.197 | 0.6976 | -2.585 to 0.1906 | 1.716 | 0.0899 | ns |
| β4 | age | 0.04201 | 0.02948 | -0.01664 to 0.1007 | 1.425 | 0.1580 | ns |
| β5 | FEV1% | -0.01032 | 0.01567 | -0.04150 to 0.02086 | 0.6584 | 0.5121 | ns |

Multiple linear regression models on HGS with calprotectin, BMI, gender, age and FEV1%.

| Parameter estimates | Variable | Estimate | Standard  error | 95% CI | \|t\| | P Value | P Value Summary |
| --- | --- | --- | --- | --- | --- | --- | --- |
| β0 | intercept | 35.51 | 6.810 | 22.01 to 49.00 | 5.214 | <0.0001 | **** |
| β1 | Calprotectin | -0.1013 | 0.02721 | -0.1552 to -0.04739 | 3.723 | 0.0003 | *** |
| β2 | BMI | -0.2135 | 0.1877 | -0.5856 to 0.1585 | 1.137 | 0.2578 | ns |
| β3 | gender | 0.3291 | 1.597 | -2.835 to 3.493 | 0.2061 | 0.8371 | ns |
| β4 | age | 0.01352 | 0.06049 | -0.1063 to 0.1334 | 0.2236 | 0.8235 | ns |
| β5 | FEV1% | 0.05480 | 0.03474 | -0.01404 to 0.1236 | 1.577 | 0.1175 | ns |

**STable 2. Mean Ct value of murine skeletal samples detected by qPCR.**

| Sample | TNF-α | IL-1β | IL-6 | IL-8 | CXCL1 | GAPDH |
| --- | --- | --- | --- | --- | --- | --- |
| Control1 | 20.10 | 19.83 | 19.45 | 21.57 | 22.04 | 14.76 |
| Control2 | 19.97 | 19.16 | 18.70 | 20.65 | 21.58 | 14.32 |
| Control3 | 18.85 | 19.19 | 18.64 | 20.79 | 21.32 | 13.83 |
| Control4 | 19.53 | 19.35 | 18.85 | 21.01 | 21.11 | 14.07 |
| Control5 | 19.45 | 19.56 | 19.24 | 21.26 | 21.53 | 14.38 |
| Control6 | 18.16 | 17.98 | 17.93 | 19.55 | 20.31 | 12.95 |
| Paquinimod(10mg/Kg)1 | 19.87 | 19.06 | 18.72 | 21.00 | 21.41 | 14.36 |
| Paquinimod(10mg/Kg)2 | 18.86 | 18.94 | 18.48 | 20.43 | 21.10 | 13.85 |
| Paquinimod(10mg/Kg)3 | 18.04 | 17.73 | 17.44 | 19.78 | 20.13 | 12.82 |
| Paquinimod(10mg/Kg)4 | 19.58 | 19.02 | 19.14 | 20.77 | 22.12 | 14.44 |
| Paquinimod(10mg/Kg)5 | 19.47 | 18.91 | 18.69 | 21.13 | 21.61 | 14.24 |
| Paquinimod(10mg/Kg)6 | 19.30 | 19.50 | 18.73 | 20.56 | 21.24 | 13.85 |
| CS1 | 15.10 | 15.34 | 15.58 | 18.17 | 18.54 | 12.96 |
| CS2 | 16.91 | 16.62 | 17.11 | 19.77 | 20.52 | 14.32 |
| CS3 | 16.78 | 16.65 | 16.31 | 18.77 | 18.71 | 14.05 |
| CS4 | 17.82 | 16.11 | 16.62 | 19.27 | 19.98 | 13.85 |
| CS5 | 16.42 | 15.99 | 16.21 | 18.82 | 19.34 | 13.52 |
| CS6 | 15.50 | 15.31 | 14.97 | 17.48 | 17.55 | 12.57 |
| CS+Paquinimod(10mg/Kg)1 | 16.02 | 16.39 | 16.04 | 18.71 | 19.22 | 12.98 |
| CS+Paquinimod(10mg/Kg)2 | 18.67 | 17.28 | 17.35 | 19.83 | 20.34 | 14.03 |
| CS+Paquinimod(10mg/Kg)3 | 17.85 | 17.58 | 18.27 | 19.87 | 20.06 | 13.77 |
| CS+Paquinimod(10mg/Kg)4 | 17.59 | 17.51 | 16.89 | 19.71 | 20.43 | 13.82 |
| CS+Paquinimod(10mg/Kg)5 | 19.45 | 18.17 | 17.69 | 20.09 | 20.73 | 14.28 |
| CS+Paquinimod(10mg/Kg)6 | 17.62 | 16.57 | 17.03 | 19.50 | 19.78 | 13.51 |
