# Supplementary figures and images for: Calprotectin Is a Circulating Biomarker and Potential Therapeutic Target for Sarcopenia in Chronic Obstructive Pulmonary Disease
Source: J Cachexia Sarcopenia Muscle. 2026 Jan 25;17(1):e70196. doi: 10.1002/jcsm.70196 (PMC12833497; doi:10.1002/jcsm.70196)

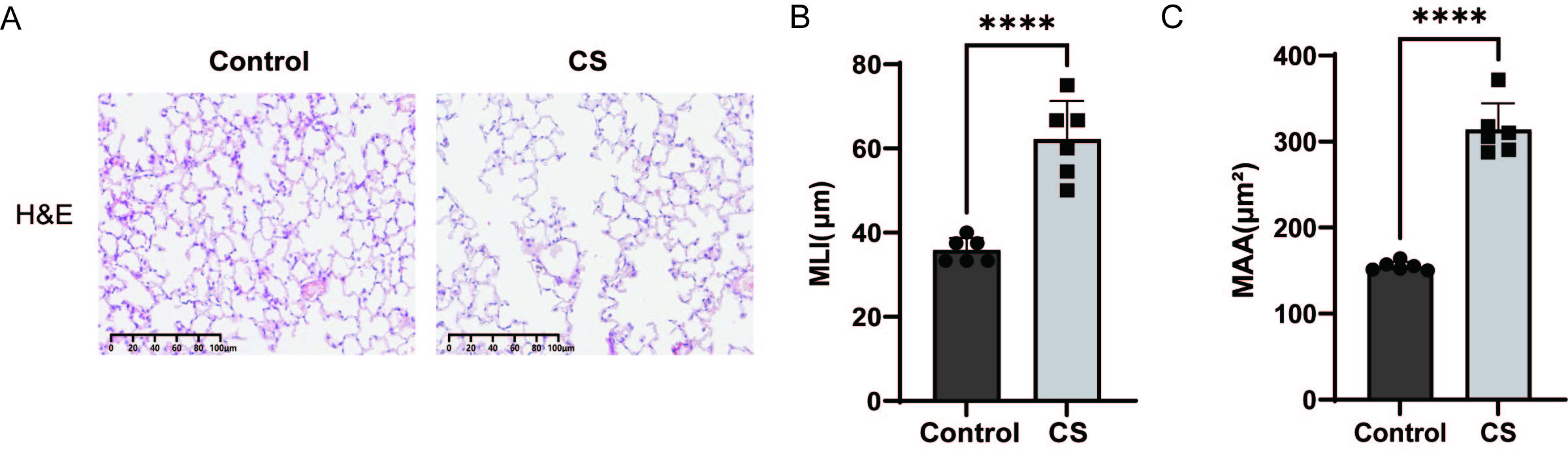

Supplement: Supplementary file 2 — Figure S1: Successful establishment of a cigarette smoke‐exposed mouse model. (A) H&E staining of the lungs. CS‐exposed mice presented enlargement of the alveolar lumen, parenchymal destruction and alveolar wall rupture. (B) Comparison of control mice and CS‐exposed mice in terms of the mean linear intercept (MLI). (C) Comparison of the mean alveolar area (MAA) between control mice and CS‐exposed mice. [file JCSM-17-e70196-s002.jpg]
